# Supplementary material for: Dawn chorus interpretation differs when using songs or calls: the Dupont’s Lark Chersophilus duponti case
Source: PeerJ. 2018 Jul 19;6:e5241. doi: 10.7717/peerj.5241 (PMC6054861; doi:10.7717/peerj.5241)
Supplement: Table S1 — Start and end time are expressed as the minutes before or later sunrise when the first and the last song or call were produced for each male. Performance time was measured as the total number of 5-min intervals that each bird was singing/calling, whereas output was estimated as the number of songs or calls uttered per male and nigh. [file peerj-06-5241-s001.pdf]

		Survey 1 (N=22)	Survey 2 (N=22)	Survey 3 (N=21)	Survey 4 (N=21)	Survey 5 (N=20)	Survey 6 (N=21)
Start time	Song	85.68 ± 6.22	63.40 ± 15.07	78.80 ± 10.23	74.29 ± 15.19	79.0 ± 11.54	76.43 ± 14.93
	Call	89.77 ± 4.49	77.5 ± 6.86	88.33 ± 8.70	87.62 ± 8.74	91.5 ± 5.87	89.52 ± 9.07
End time	Song	12.04 ± 12.87	13.86 ± 7.70	13.81 ± 11.60	15.48 ± 12.24	13.5 ± 16.39	15.24 ± 13.74
	Call	-1.36 ± 7.27	-4.32 ± 12.28	-0.95 ± 5.62	-2.38 ± 7.00	-2.75 ± 5.25	-2.14 ± 5.14
Performance time	Song	14.50 ± 3.38	10.54 ± 3.36	12.33 ± 1.71	11.38 ± 2.86	12.5 ± 2.68	12.52 ± 3.88
	Call	13.18 ± 2.74	9.28 ± 2.17	7.86 ± 2.35	8.43 ± 1.83	9.05 ± 2.39	9.24 ± 2.16
Output	Song	103.73 ± 48.69	116.31 ± 49.79	128.38 ± 46.65	125.29 ± 55.23	126.20 ± 40.80	130.38 ± 41.23
	Call	96.86 ± 59.58	59.18 ± 28.26	44.43 ± 26.86	48.76 ± 25.56	44.05 ± 23.20	45.71 ± 19.86
