# Supplementary material for: Dawn chorus interpretation differs when using songs or calls: the Dupont’s Lark Chersophilus duponti case
Source: PeerJ. 2018 Jul 19;6:e5241. doi: 10.7717/peerj.5241 (PMC6054861; doi:10.7717/peerj.5241)
Supplement: Table S2 [file peerj-06-5241-s002.pdf]

	R	R^2	P
Survey 1	- 0.9204	0.8472	< 0.0001
Survey 2	- 0.4681	0.2191	0.0280
Survey 3	0.2911	0.0847	0.2004
Survey 4	0.0580	0.0034	0.8028
Survey 5	0.3973	0.1579	0.0828
Survey 6	0.2668	0.0712	0.2423
